# One-to-one care routines and compliance with the national professional recommendation on continuous intrapartum support in Norway: A national survey

**DOI:** 10.18332/ejm/110064

**Published:** 2019-07-25

**Authors:** Birgitte G. Bjerga, Mari Risa, Ellen Blix, Aase S. Devold Pay

**Affiliations:** 1 Department of Obstetrics and Gynecology, Oslo University Hospital, Oslo, Norway; 2 Department of Obstetrics and Gynecology, Stavanger University Hospital, Stavanger, Norway; 3 Faculty of Health Sciences, Oslo Metropolitan University, Oslo, Norway

**Keywords:** one-to-one care, continuity of care, clinical recommendation, labor, questionnaire

## Abstract

**INTRODUCTION:**

In 2010, the Norwegian Directorate of Health introduced the guideline ‘Safe maternity services – quality standards for maternal care’. These standards include adequate staffing with health care personnel for birth units to ensure responsible monitoring and treatment. Birth units are to follow the professional recommendation that every woman has a midwife present during established labor. This study presents data from birth units on compliance with the national recommendation for one-to-one care during labor.

**METHODS:**

A web-based questionnaire was emailed to chief midwives of all birth units in Norway (n=48) in May 2018. The questionnaire contained a total of nine multiple-choice, scaled-response-format, and free-text questions.

**RESULTS:**

The questionnaire response rate was 100%. All birth units reported that they offered women one-to-one care during labor to a large extent. Sixty-five per cent of the birth units had procedures to ensure that midwives were present during established labor. Deviations from the recommendation were recorded in one-fourth of birth units. Thirty-eight per cent of respondents reported that staff training had been provided; 56% of birth units stated that the recommendation led to an increased presence of midwives during labor. Financial constraints (35%) and difficulty of compliance (27%) were cited as obstacles to meeting the recommendation for one-to-one care during labor.

**CONCLUSIONS:**

The majority of birth units reported that they follow the recommendation for one-to-one care during established labor, but compliance with this recommendation in practice remains unclear. Areas of improvement relate to routines describing the presence of midwives during labor, registration of deviations, and staff training in one-to-one care.

## INTRODUCTION

In 2010, the Norwegian Directorate of Health introduced the guideline ‘Safe maternity services – quality standards for maternity care’^1^. These quality standards include adequate staffing with health care personnel in birth units to ensure responsible monitoring and treatment. Birth units are to follow professional recommendations stating that all women in labor should have midwives present at all times during the active stage of labor (one-to-one intrapartum support). The Norwegian Directorate of Health concluded that good evidence supports this standard of supportive one-to-one care, and that it will have several positive effects for women with low-risk and high-risk pregnancies.

One-to-one intrapartum support enables midwives to provide emotional support, information, and guidance. This level of support may promote normal birth by increasing the delivering woman’s sense of control and ability to cope while reducing the need for childbirth interventions^2^. Women with access to the continuous presence of a midwife during labor are more likely to give birth spontaneously, reducing the risk of instrumental vaginal delivery and caesarean section, and improving babies’ Apgar scores. A midwife’s presence can affect a laboring woman’s choice of pain relief and be an important factor in the quality of a woman’s birth experience^2-5^.

In 2017, the Ministry of Health and Care Services issued an assignment document to regional health authorities, which required these authorities to document compliance with the one-to-one principle^6^. In this document, birth units were to “ensure that incidences of non-compliance with the recommendation for women to have a midwife present as early as possible during established labor until the birth of the child, and whether this was documented in the health authorities’ deviation systems, where results would be used for quality improvement”.

The guidelines from the Directorate of Health serve as advisory instructions in health care services to achieve sound professional procedures, and are used to ensure good quality and appropriate prioritization^7^. Regional health authorities and district health trusts are responsible for ensuring that services are performed in a responsible manner, and that national guidelines are put into practice. The purpose of this study was to obtain information from Norwegian birth units on the implementation for one-to-one care, and to determine whether birth units register breaches of the one-to-one principle in maternity care.

## METHODS

An open invitation to answer a web-based questionnaire was emailed to chief midwives of all birth units in Norway (n=48) in May 2018. This email included information about the study and a link to the questionnaire. The chief midwives consented to their participation by answering the questionnaire. One or two reminders were sent, as needed. All responses were received in August 2018.

The questionnaire had two parts: ‘Background information’ and ‘One-to-one continuous presence during established labor’. It contained a total of nine multiple-choice, scaled-response-format, and free-text questions. Scaled responses were structured according to a 6-point Likert scale ranging (1 = ‘to a very small extent/seldom’ to 6 = ‘to a very large extent/always’). The questions on one-to-one continuous presence during established labor are shown in Table 1. The questionnaire was piloted and tested among midwives at Oslo University Hospital during the spring 2018.

**Table 1:** The questions on one-to-one continuous presence during established labor

Does the birth unit have written procedures to ensure the continuous presence of a midwife during labor? Yes /No /Don’t know.
To what extent are women in established labor offered one-to-one intrapartum support during established labor? Scaled response from 1 to 6.
Does the birth unit have internal documentation of the number of women in labor provided with one-to-one intrapartum support? Yes /No /Don’t know.
What is done if a woman does not have access to the presence of a midwife during labor? A deviation report is always sent /A deviation report is sent now and then or seldom /No routines for this circumstance /Other (free text).
Has the labor and delivery unit provided its staff with training on the continuous presence of midwives during active stage of labor? Yes /No
Has the professional recommendation for one-to-one intrapartum support led to changes? Presence during labor /Staff /Staff training /Other (free text).
Are there any obstacles to compliance with the professional recommendation for one-to-one intrapartum continuous support? Lack of financing /Patient’s wishes /Unnecessarily stringent recommendation /Difficult to fulfil recommendation /Other (free text).

The Norwegian Social Science Data Services approved this study (reference no. 60699; 22 May 2018). According to the Norwegian Health Research Act, surveys for studying attitudes and practices among healthcare professionals fall outside the remit of the Research Ethics Committees. Therefore, this project did not require approval from a Research Ethics Committee.

Data were analyzed using SPSS version 24 (IBM Corporation, Armonk, NY, USA). Descriptive analyses, including frequency calculation and cross table production, were then performed.

## RESULTS

All (48/48, 100%) respondents filled in the questionnaire. Table 2 shows the number of respondents by level of obstetric care and number of deliveries in 2018.

**Table 2:** Number of respondents by level of obstetric care and deliveries in 2018

*Number of deliveries*	*Specialized obstetric units (N=17)*	*Obstetric units in local hospital (N=22)*	*Freestanding midwifery led units (N=6)*	*Alongside midwifery led units (N=3)*	*Total (N=48)*
	*n (%)*	*n (%)*	*n (%)*	*n (%)*	*n (%)*
<500	0 (0)	14 (64)	6 (100)	0 (0)	20 (42)
500–999	1 (6)	6 (27)	0 (0)	2 (67)	9 (19)
1000–1999	8 (47)	2 (9)	0 (0)	1 (33)	11 (23)
≥2000	8 (47)	0 (0)	0 (0)	0 (0)	8 (16)

Two-thirds (31/48, 65%) of the birth units had written procedures to ensure that midwives were present during established labor. All 48 units indicated that they offered women one-to-one care during labor to: a large extent, a very large extent, or always (Table 3). Seventeen (17/48, 35%) of the birth units reported to have documentation of the number of women in labor provided with this service.

**Table 3:** Extent to which women in labor are offered continuous midwife support during established labor, reported by chief midwives of Norwegian birth units in 2018

*Continuous support by midwives during labor^a^*	*Specialized obstetric units (N=17)*	*Obstetric units in local hospital (N=22)*	*Freestanding midwifery led units (N=6)*	*Alongside midwifery led units (N=3)*	*Total (N=48)*
	*n (%)*	*n (%)*	*n (%)*	*n (%)*	*n (%)*
6	8 (47)	18 (82)	6 (100)	2 (67)	34 (71)
5	8 (47)	2 (9)	0 (0)	1 (33)	11 (23)
4	1 (6)	2 (9)	0 (0)	0 (0)	3 (6)
3	0 (0)	0 (0)	0 (0)	0 (0)	0 (0)
2	0 (0)	0 (0)	0 (0)	0 (0)	0 (0)
1	0 (0)	0 (0)	0 (0)	0 (0)	0 (0)

a Based on a 6-point Likert scale: 1 = ‘very small extent/seldom’ to 6 = ‘very large extent/always’.

Forty-six birth units responded to the question regarding whether the failure to offer a woman in labor the presence of a midwife was reported to the department’s deviation system. The majority (27/46, 59%) of respondents indicated that deviation reports were sent now-and-then or seldom. Twelve (12/46, 26%) birth units indicated that deviations were always reported, whereas seven (7/46, 15%) units did not have routines for the submission of such reports. Eighteen (18/48, 38%) units reported that they had provided staff training in their wards on the presence of midwives during labor.

The recommendation for one-to-one care led to increases (self-reported) in the presence of midwives during labor in 27 (27/48, 56%) birth units. Nine (9/48, 19%) units stated that the recommendations led to changes in training and education. Six (6/48, 13%) units stated that the recommendations led to increased staff. In a free-text response, one respondent wrote: ‘The recommendation for more frequent presence has raised awareness of the presence of midwives during the active stage of labor’. Another stated that they had initiated projects and improvement measures directly aimed at increasing focus on the presence of midwives during established labor.

**Figure 1:**
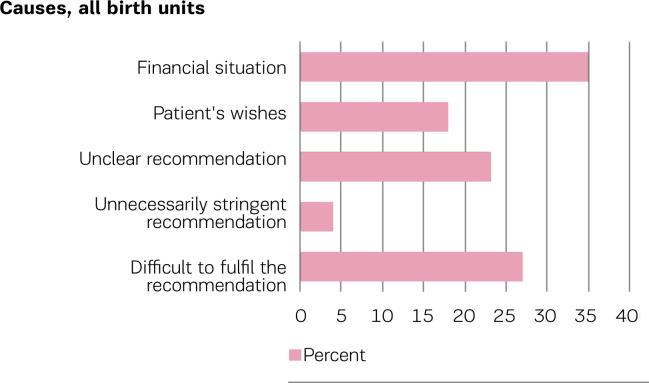
Greatest obstacles to compliance with the national clinical recommendation for one-to-one care during labor, reported by chief midwives of Norwegian birth units in 2018 (n=48)

Figure 1 shows respondents’ perceived greatest obstacles to compliance with the professional recommendation for one-to-one care. The lack of financing was noted as a reason by 35% of respondents, and 27% stated that compliance with the recommendation was difficult.

## DISCUSSION

The findings of this study indicate that the Norwegian Directorate of Health’s recommendation for one-to-one care during established labor has had an impact on reported changes in the presence of midwives, but that challenges associated with routines that describe one-to-one care during established labor, the registration of deviations, and staff training, remain.

The majority of birth units have introduced written procedures to ensure that midwives are present during established labor. In a 2015 study examining the implementation of the Norwegian Directorate of Health’s quality standards for maternity institutions, about half of the institutions did not have written criteria covering this practice^8^. Written procedures are essential for the provision of good health care services^9^. Procedures should provide support for health care personnel in their daily work and contribute toward a high and predictable quality of services.

All birth units stated that they provided one-to-one care to at least a large extent, and 35% of units had overviews of the number of women in labor that did have midwives present during established labor. Maternity services must focus on planning midwife care adequately in advance to ensure safe care for mothers and babies^10^. The National Institute for Health and Care Excellence has produced a step-by-step guide for organizations to determine the number of midwives required^11^. Using local records to help predict requirements, hospital boards can constantly assess staffing levels to ensure that mothers in labor receive one-to-one care.

The 2017 assignment document for regional health authorities included the requirement that health trusts document incidences of non-compliance with the recommendation for the presence of a midwife, and to report these incidences within the deviation system^6^. The purpose of this requirement was not to implement a procedure of reporting to the Ministry, but rather to ensure that deviations from the recommendation are used for health authorities’ quality improvement efforts^12^. One-fourth of maternity institutions stated that deviation reports were always sent, whereas more than half reported that deviation reports were sent occasionally or seldom.

The documentation of deviations takes time, and when deviations occur because midwives have too little time, such registration may not occur. This practice must be facilitated by management; health trusts must have user-friendly systems that facilitate such registration.

### Limitations and strengths

The study has some limitations. The respondents were chief midwives; thus, the responses reflect these professionals’ subjective views, rather than general views of the unit staff. Furthermore, the study did not examine whether all midwives in the birth units were aware of the recommendation for one-to-one care.

Certain questionnaire items allowed for some degree of judgment and discretion. Some questions were not answered by all respondents, perhaps because the individuals filling in the questionnaire had insufficient information on the subject. In addition, the questionnaire did not sufficiently address whether procedures are followed. Nevertheless, we believe that this study offers important information regarding the routines and structure of maternity institutions in Norway, and potential measures for quality improvement. The strength of this study is that it included data from all birth units in Norway.

## CONCLUSIONS

The recommendation for one-to-one care during established labor has led to reported changes in maternity care. This study shows that birth units generally follow national recommendations, but that challenges associated with routines that describe the presence of midwives during the established stage of labor, registration of incidents of non-compliance with the recommendation, and staff training, remain. It is important for employers to be aware of the degree of compliance with quality standards, to guide the design of improvement measures and assessment of the effects of their implementation.
